# Neuro-immune interactions underlying autoimmune diseases: insights from brain imaging data

**DOI:** 10.1186/s13041-026-01292-4

**Published:** 2026-03-19

**Authors:** Junxiang Gu, Zixuan Xing, Jian Wang, Yalong He, Juan Li, Cong Wu, Wenlei Kuo, Xianxia Yan, Wei Lin, Tao Chen

**Affiliations:** 1 Department of Neurosurgery, Xijing Hospital, Fourth Military Medical University, Shaanxi, China; 2 Department of Anatomy, Histology and Embryology and K.K. Leung Brain Research Centre, Fourth Military Medical University, Shaanxi, China; 3 Department of Neurosurgery, The Second Affiliated Hospital of Xi’an Jiaotong University, Shaanxi, China; 4 Department of Aviation Medicine, Xijing Hospital, Fourth Military Medical University, Shaanxi, China

**Keywords:** Migraine, AIDs, IDPs, CNS

## Abstract

Autoimmune diseases (AIDs) result from intricate interactions among genetic, environmental and immune factors, with emerging evidence underscoring the role of the central nervous system (CNS) in their pathogenesis. In this study, we utilized two-sample Mendelian randomization to systematically explore the causal relationships between 3,935 brain imaging-derived phenotypes (IDPs) and eight AIDs, including atopic dermatitis, inflammatory bowel disease, lupus erythematosus, multiple sclerosis, myasthenia gravis, psoriasis, rheumatoid arthritis and type 1 diabetes. We identified nine IDPs with causal associations to eight representative AIDs. Mediation analysis uncovered eight potential immune cells or inflammatory factors bridging IDPs and AIDs. These findings provide compelling evidence for the CNS’s involvement in autoimmune progresses through neuro-immune pathways and underscore potential diagnostic and therapeutic targets within the neuro-immune axis. This study introduces a novel framework for investigating interdisciplinary interventions that target CNS-immune interactions in the context of AIDs.

**Supplementary Information:**

The online version contains supplementary material available at 10.1186/s13041-026-01292-4.

## Introduction

Autoimmune diseases (AIDs) constitute a diverse group of disorders characterized abnormal immune responses targeting the body’s own tissues, often resulting in chronic inflammation and progressive tissue damage [1–3]. They represent a significant global health burden due to their high prevalence, mortality rates and socioeconomic impact [4]. This highlights the need for innovative and interdisciplinary approaches to understanding and managing these diseases.

Previous studies have demonstrated the regulatory role of the central nervous system (CNS) in peripheral immunity [5]. They prompt the pathways by which the CNS modulates immune responses and identify key brain regions, such as the prefrontal and insular cortices, as major players in this process. Experimental animal studies have confirmed the CNS’s critical role in regulating immune responses, with notable effects on inflammation in peripheral organs like the lungs and abdominal cavity [6, 7]. This evidence suggests the existence of a neuro-immune communication that may influence the development and progression of AIDs. However, the specific brain regions or functional connections involved in this regulation remain poorly understood.

In tandem with these findings, advancements in neuroimaging analysis have enabled the identification of brain imaging-derived phenotypes (IDPs), which typically include the volume of specific brain structures, the connectivity between brain regions, and the diffusion characteristics of white-matter tracts [8, 9].To date, over 3,935 brain IDPs have been identified [10]. Emerging studies have explored their causal relationships with neurological traits and certain systemic diseases, particularly neuropsychiatric disorders [11, 12]. Indeed, neuroimaging studies have shown that AIDs are often accompanied by significant alterations in IDPs. Multiple sclerosis (MS), the most extensively studied AID, exhibits reduced white matter integrity as evidenced by diffusion tensor imaging (DTI) [13] and diminished connectivity in the default mode network (DMN), via resting-state functional MRI (rfMRI) [14, 15]. Other systemic AIDs also show distinctive IDPs patterns: rheumatoid arthritis (RA) is associated with reduced cortical thickness and gray matter volume [16], lupus erythematosus (LE) demonstrates altered DMN connectivity [17], and Inflammatory bowel disease (IBD) is linked to abnormal functional connectivity in the insula, cingulate cortex and limbic system [18, 19]. Remarkably, certain IDPs, such as cortical thickness and specific rfMRI connectivity patterns, have been proposed as biomarkers for predicting disease severity and progression [20, 21].

However, most of these studies are observational, leaving the causal relationships between IDPs changes and AIDs unclear. It remains uncertain whether these brain phenotype alterations are consequences of AIDs or whether they serve as causal factors in the pathogenesis of these diseases. Furthermore, immune cells and inflammatory mediators play a pivotal role in the development and progression of AIDs [22, 23]. They not only influence CNS function but are also regulated by it. Nevertheless, their potential role as intermediaries linking brain phenotypes to AIDs has yet to be systematically explored.

To address these issues, we employ Mendelian randomization (MR), a method that uses genetic variants as instrumental variables to infer causality, and reduce confounding bias. By integrating IDPs and AIDs with genome-wide association study (GWAS) data, we aim to elucidate the causal relationships between 3,935 IDPs and eight AIDs affecting different systems: atopic dermatitis (AD), IBD, lupus erythematosus (LE), MS, myasthenia gravis (MG), psoriasis (PSO), RA and type 1 diabetes (T1D). Additionally, we aim to explore the mediating roles of immune cells and inflammatory factors in these complex relationships. This study will deepen our understanding of neuro-immune communications in AIDs and provide a comprehensive “map” of brain structures and functional connections involved in regulating these diseases. By doing so, it will highlight the CNS as a crucial yet often overlooked contributor to autoimmunity, and pave the way for novel diagnostic and therapeutic strategies for AIDs by targeting neural pathways.

## Methods

### Study design

We used a two-sample Mendelian randomization (MR) approach to investigate causal relationships between 3,935 brain IDPs and eight AIDs: AD, IBD, LE, MS, MG, PSO, RA and T1D. Summary-level GWAS data were obtained from large, well-characterized cohorts. Mediation analyses were further conducted to explore the potential roles of immune cells and inflammatory factors as mediators in these causal pathways.

Data sources.

### Brain imaging-derived phenotypes and autoimmune diseases

GWAS summary statistics for 3935 IDPs were obtained from the UK Biobank cohort containing data from 33,224 subjects [10]. These phenotypes encompass structural and functional brain imaging data derived from magnetic resonance imaging (MRI), including measures such as cortical thickness, white matter integrity, and functional connectivity. We provide all the corresponding regions and brain functions related to rfMRI connectivity (Additional file 1: Table S1). To minimize sample overlap, summary statistics for eight AIDs were retrieved from the FinnGen release 11. The psoriasis GWAS dataset consists of 11,479 cases and 437,420 controls. The myasthenia gravis GWAS dataset consists of 504 cases and 449,570 controls. The multiple sclerosis GWAS dataset consists of 2620 cases and 449,770 controls. The lupus erythematosus GWAS dataset consists of 777 cases and 423,041 controls. The inflammatory bowel disease GWAS dataset consists of 9759 cases and 443,974 controls. The atopic eczema GWAS dataset consists of 26,905 cases and 394,476 controls. The type 1 diabetes GWAS dataset consists of 109 cases and 364,434 controls. The rheumatoid arthritis GWAS dataset consists of 14,818 cases and 287,796 controls (Additional file 1: Table S2).

### Inflammatory factors and immune cell signatures

To minimize sample overlap, we utilized the genome-wide associations of mediators that were not from the UKB and FinnGen. GWAS data for 91 inflammatory factors were obtained from Zhao et al. [24]. The 91 inflammatory factors were measured using the Olink Target platform in 14,824 participants. The GWAS data for 731 immune cell signatures were derived from the SardiNIA project, with data collected from 3757 Sardinians [25].

Mendelian randomization analysis.

### Genetic variants serving as instruments

MR infers the causal relationship between exposure and outcome by using genetic variations as instrumental variables (IVs). We use the F-statistic to measure the strength of the IVs. The standard rule for a strong instrument is that the F-statistic of the instrument-exposure association should exceed 10. The calculation is as follows: F = *β*² / SE², where *β* represents the estimated genetic effect on the exposure, and SE represents the standard error of *β*. For each single nucleotide polymorphism (SNP), except for T1D and MG, all other AIDs were considered as instruments with trait association *p* < 5 × 10^− 6^. For IDPs, T1D, MG, immune cells, and inflammatory factors, a more relaxed threshold of *p* < 1 × 10^− 5^ was used due to limited available SNPs. We excluded associated SNPs at linkage disequilibrium thresholds of r^2^ > 0.001 and distance > 10,000 kb, retaining the SNPs with the strongest effect.

### Forward MR analysis

We performed forward MR to evaluate the causal effects of IDPs on each autoimmune disease using five MR methods: Inverse variance weighted (IVW), MR Egger, Weighted median, Simple mode and Weighted mode. To ensure robustness, we excluded: (1) IDPs with evidence of horizontal pleiotropy (MR-Egger intercept *p* < 0.05 or MR PRESSO global test *p* < 0.05); (2) Associations with heterogeneity (Cochran’s Q-test *p* < 0.05); (3) IDPs with inconsistent effect direction across methods; (4) IDPs with IVW BH-adjusted *p* > 0.05.

### Reverse MR analysis

Reverse MR analysis was performed on the prioritized IDPs to rule out reverse causation. IDPs with IVW *p* < 0.05 in reverse MR were excluded for subsequent mediation MR analyses.

### Mediation MR analysis

To explore potential mediators, the following criteria should be applied: Step 1: Two-sample MR was performed between immune cells/inflammatory factors and AIDs to identify mediators causally associated with AIDs (IVW *p* < 0.05). Step 2: Two-sample MR was conducted between the remaining IDPs and the identified immune cells/inflammatory factors to establish causal effects of IDPs on these mediators. Step 3: Mediation MR analysis was applied to quantify the proportion of causal effect of IDPs on AIDs that is mediated through immune cells or inflammatory factors.

### Statistical analyses

All analyses were conducted in R (version 4.4.1) using the ‘TwoSampleMR’ packages. Key assumptions of MR (relevance, independence, and exclusion restriction) were assessed to validate the robustness of causal inference.

## Results

### Overview of the study

**Fig. 1:**
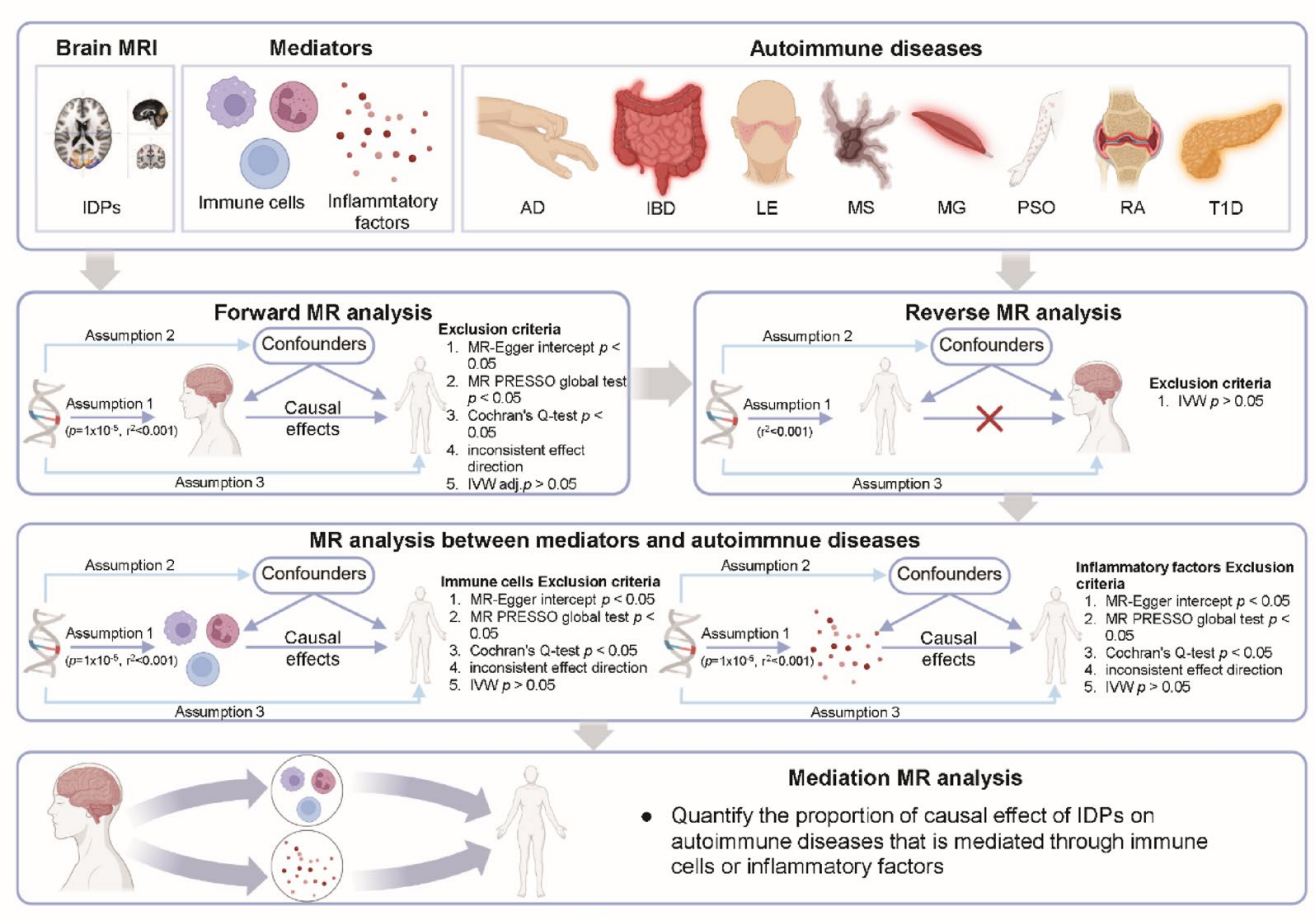
Overview of the study design. In brief, 3935 brain MRI phenotypes, 8 autoimmune diseases, immune cells, and inflammatory factors were used for MR analysis. Forward MR analysis was performed first, and then the obtained results were subjected to reverse MR analysis. Mediation analysis was further performed. MRI, Magnetic resonance imaging; AD, atopic dermatitis; IBD, inflammatory bowel disease; LE, lupus erythematosus; MS, multiple sclerosis; MG, myasthenia gravis; PSO, psoriasis; RA, rheumatoid arthritis; T1D, type 1 diabetes; IDPs, imaging-derived phenotypes; MR, Mendelian randomization; IVW, Inverse-Variance Weighted

To explore the potential causal relationship and mediator effects between IDPs and AIDs, we performed two-sample Mendelian randomization analysis using summary statistics of GWAS of 3935 IDPs and 8 AIDs from UK Biobank. We also investigated the potential role of immune cells and inflammatory factors as mediators. Detailed datasets of IDPs, AIDs and mediators are available in the original publications and summarized in Additional file 1 (Table S2). An overview of the study design is presented in Fig. 1. To meet the basic assumptions of MR, we rigorously controlled the quality of instrumental variables (IVs) used. To assess the robustness of our MR inference, we conducted a sequential sensitivity analysis (Fig. 1). Additionally, we performed a reverse MR analysis to confirm the absence of reverse causality prior to conducting the mediation analysis. Collectively, these evidences indicate that our selected IVs are appropriate and that both the causal relationship and mediation analysis in the MR estimation are reliable.

### Causal associations between IDPs and eight AIDs

**Fig. 2:**
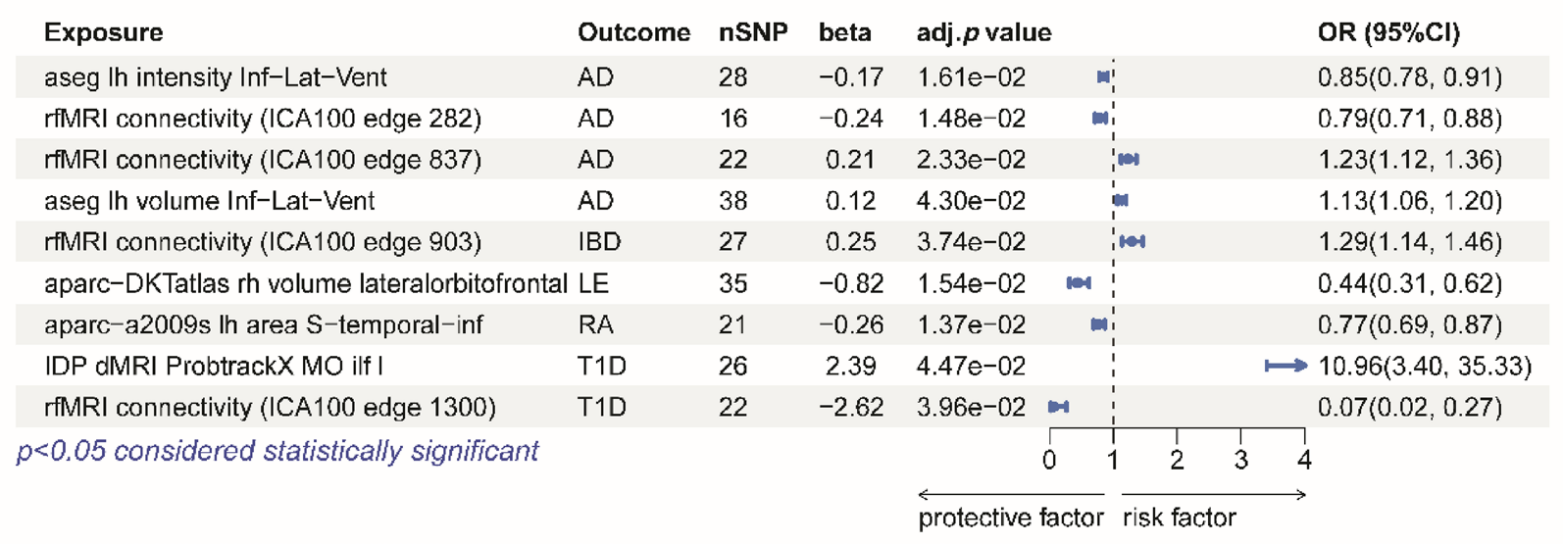
The forest plot shows the IDPs that have a causal relationship with AD, IBD, LE, RA and T1D. Forest plots show significant causal relationships estimated using the MR-IVW method. For the MR-IVW method, BH-adjusted *P* < 0.05 was considered significant. OR represent the effect size of a 1 standard deviation change in the mean IDPs phenotype on the risk of autoimmune disease, and error bars represent 95% confidence intervals. AD, atopic dermatitis; IBD, inflammatory bowel disease; LE, lupus erythematosus; RA, rheumatoid arthritis; T1D, type 1 diabetes; OR, odds ratio; SNP, single nucleotide polymorphism; CI, Confidence interval

Through MR analysis, we identified nine IDPs that are causally associated with eight representative AIDs (Additional file 1: Table S3, S4). Specifically, four IDPs were associated with AD, with two protective factors and two risk factors; one risk IDP was associated with IBD; one protective IDP was associated with LE; none of IDPs were associated with MS, MG, and PSO; one protective IDP was associated with RA; two IDPs were associated with T1D, with one protective factor and one risk factor (Fig. 2). The brain regions most frequently implicated across all conditions were the frontal lobe and temporal lobe.

### Causal associations between immune cells and eight AIDs

**Fig. 3:**
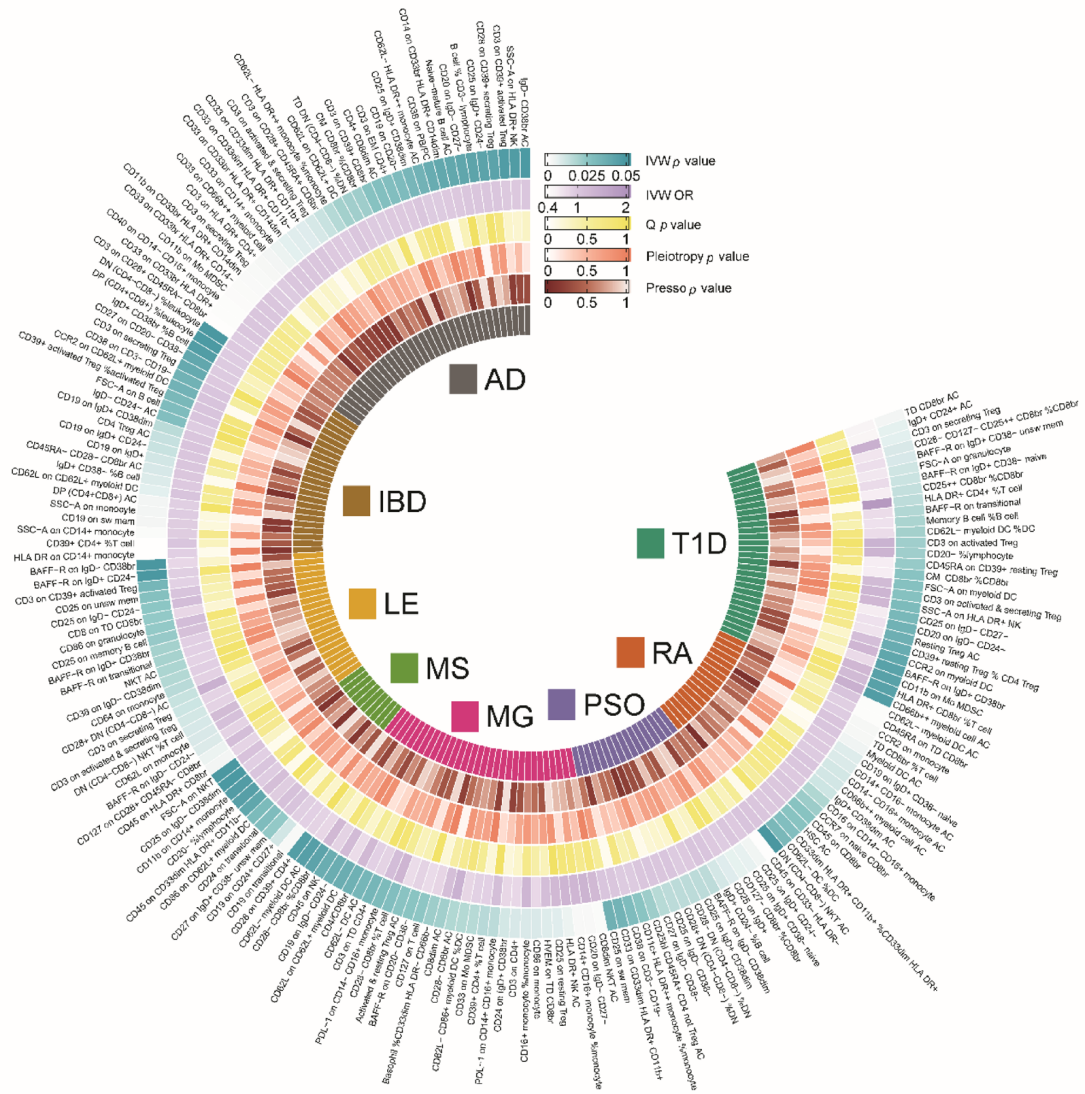
The causal relationship between immune cells and 8 autoimmune diseases. Heatmaps showing significant causal relationships estimated using the MR-IVW method. For the MR-IVW method, *P* < 0.05 was considered significant. OR represent the effect size of a 1 standard deviation change in the mean immune cells on the risk of autoimmune disease, and error bars represent 95% confidence intervals. AD, atopic dermatitis; IBD, inflammatory bowel disease; LE, lupus erythematosus; MS, multiple sclerosis; MG, myasthenia gravis; PSO, psoriasis; RA, rheumatoid arthritis; T1D, type 1 diabetes; OR, odds ratio; IVW, Inverse-Variance Weighted

We identified immune cells with causal relationships to the eight AIDs (Fig. 3, Additional file 1: Table S5). AD was linked to 35 immune cell types, with 18 protective and 17 risk-related. IBD was linked to 23 immune cell types, with 12 protective and 11 risk-related. LE was linked to 21 immune cell types, with 13 protective and 8 risk-related. MS was linked to 10 immune cell types, with five protective and five risk-related. MG was linked to 31 immune cell types, with 16 protective and 15 risk-related. PSO was linked to 17 immune cell types, with seven protective and 10 risk-related. RA was linked to 17 immune cell types, with seven protective and 10 risk-related. Finally, T1D was linked to 28 immune cell types, with 16 protective and 12 risk-related.

### Causal associations between inflammatory factors and eight AIDs

**Fig. 4:**
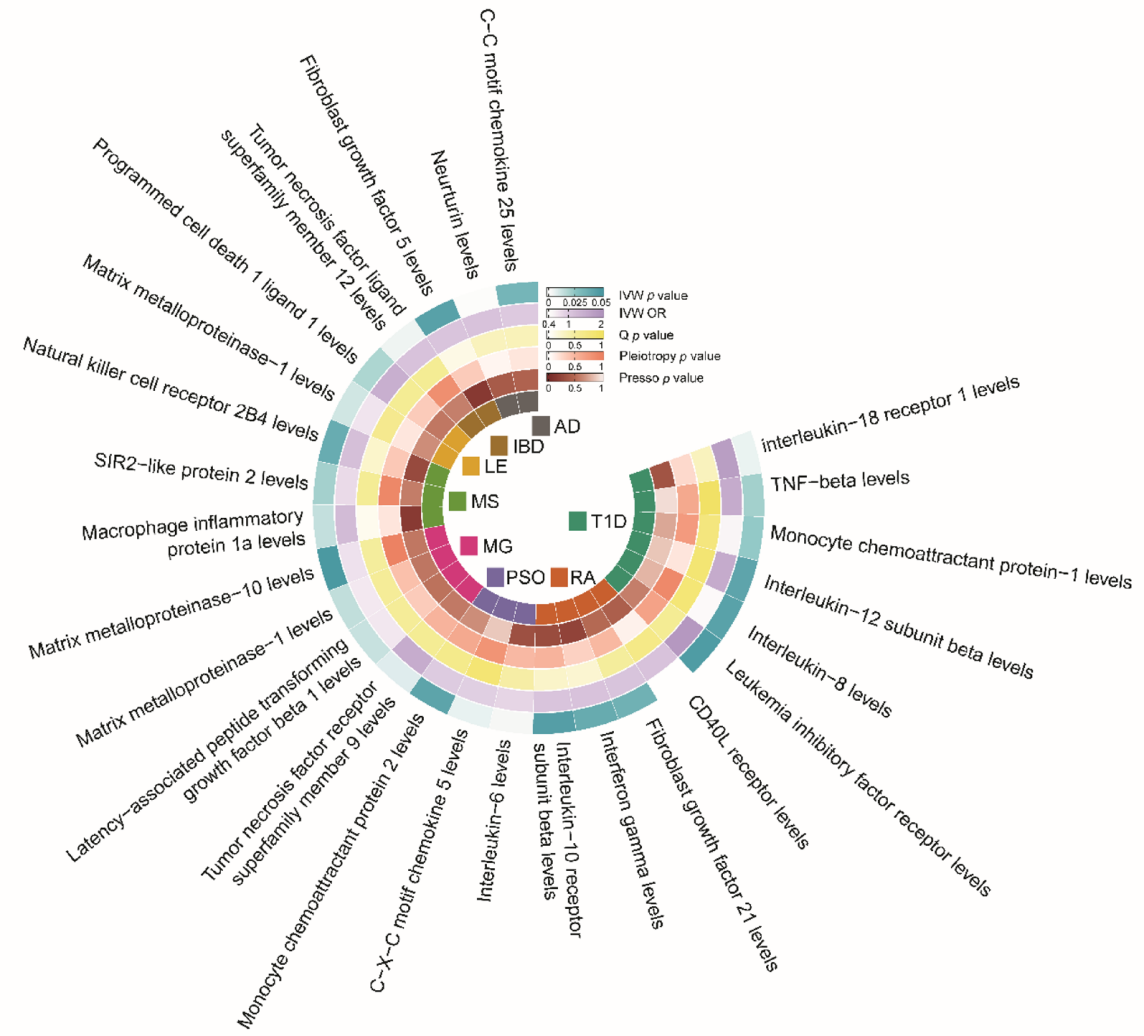
The causal relationship between inflammation factors and 8 autoimmune diseases. Heatmaps showing significant causal relationships estimated using the MR-IVW method. For the MR-IVW method, *P* < 0.05 was considered significant. OR represent the effect size of a 1 standard deviation change in the mean inflammation factors on the risk of autoimmune disease, and error bars represent 95% confidence intervals. AD, atopic dermatitis; IBD, inflammatory bowel disease; LE, lupus erythematosus; MS, multiple sclerosis; MG, myasthenia gravis; PSO, psoriasis; RA, rheumatoid arthritis; T1D, type 1 diabetes; OR, odds ratio; IVW, Inverse-Variance Weighted

We identified inflammatory factors causally associated with the eight AIDs (Fig. 4, Additional file 1: Table S6). AD was linked to two inflammatory factors, with one protective and one risk-related. IBD was linked to two risk-related factors. LE was linked to one protective and one risk factor. MS was linked to three inflammatory factors, two of which were risk-related. MG was linked to four inflammatory factors, one of which were risk-related. PSO was linked to three inflammatory factors, all of which were protective-related. RA was linked to four inflammatory factors, all of which were risk-related. Lastly, T1D was linked to six inflammatory factors, four of which were risk-related.

### Mediating effects of immune cells and inflammatory factors in IDP-autoimmune disease associations

We explored the mediating effects of immune cells and inflammatory factors in the causal pathways between IDPs and AIDs (Additional file 1: Table S7, S8). Based on the criteria described in Fig. 1, eight potential IDPs-immune cell/inflammatory factor-AIDs pathways were identified (Additional file 1: Table S9).

**Fig. 5:**
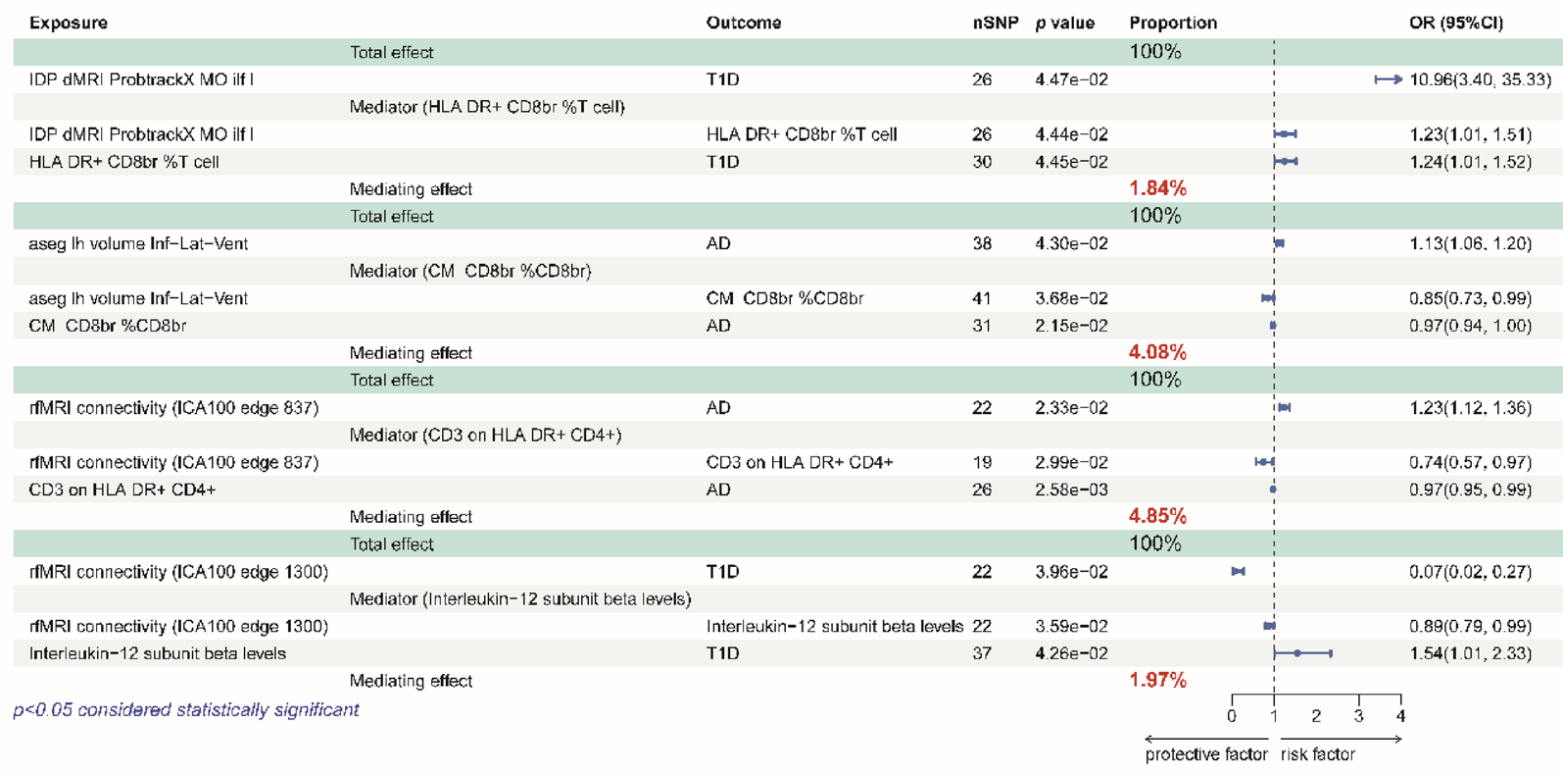
Mendelian randomization estimates of proportions mediated by mediators in the causal association between IDPs and AIDs. Forest plots show significant causal relationships estimated using the MR-IVW method. For the MR-IVW method, *P* < 0.05 was considered significant. OR represent the effect size of 1 standard deviation change in the mean exposure on the risk of the outcome, and error bars represent 95% confidence intervals. AD, atopic dermatitis; T1D, type 1 diabetes; OR, odds ratio; SNP, single nucleotide polymorphism; CI, Confidence interval

In T1D, percentage of HLA-DR+ CD8br T cells among total T cells mediated the causal pathway from “IDP dMRI ProbtrackX MO ilf l” to T1D, accounting for 1.84% of the total effect; interleukin-12 subunit beta levels mediated the causal pathway from IDP “rfMRI connectivity [Independent Component Analysis (ICA)100 edge 1300]” to T1D, accounting for 1.97% of the total effect. In AD, percentage of central memory CD8 bright T cells among total CD8 bright T cells mediated the causal pathway from “aseg lh volume Inf-Lat-Vent” to AD, accounting for 4.08% of the total effect; CD3 on HLA DR+ CD4 + mediated the causal pathway from “rfMRI connectivity (ICA100 edge 837)” to AD, accounting for 4.85% of the total effect (Fig. 5).

## Discussion

In this study, we employed a comprehensive Mendelian randomization (MR) approach to explore causal relationships between 3,935 brain IDPs and eight AIDs, and investigated the potential mediating roles of 731 immune cell phenotypes and 91 inflammatory factors in these causal relationships. We identified nine of IDPs that are causally associated with eight representative AIDs. Among these IDPs, both protective factors and risk factors were identified. The brain regions most frequently implicated across all AIDs were the frontal lobe and temporal lobe. Mediation analysis revealed eight potential cells/inflammatory factors linking IDPs and AIDs.

Our observations of the significant IDPs for each disease revealed no common IDP shared across all eight AIDs. However, upon further analysis, we found that the frontal lobe and temporal lobe were frequently implicated, suggesting that these structures may play crucial roles in the neuro-immune axis, although their effects may differ within the specific connectivity networks associated with various AIDs.

The frontal lobe, as a core region for executive functions, primarily engages in emotion regulation, advanced cognition and behavioral control [26]. Dysfunction in this region has been implicated in AIDs. For instance, in systemic LE (SLE) patients, reduced prefrontal function is positively correlated with elevated levels of chronic pro-inflammatory factors, such as IL-6 and TNF-α [27], potentially creating a vicious cycle of neuroinflammation and immune dysregulation. Studies on MS have shown that the expansion of frontal white matter lesions significantly correlates with disease progression and cognitive decline [28]. Our results also showed that the relevant functional connectivity of the frontal lobe were protective factors for MS. This suggests that the frontal lobe might be responsible for the spread of neuroinflammation, which can directly or indirectly lead to further systemic immune dysregulation.

Previous studies indicate that epilepsy, especially temporal lobe epilepsy, often co-occurs with systemic AIDs such as T1D [29–31] and LE [32]. Our results also showed that the temporal lobe and its area were risk factors for LE, and the functional connectivity between the temporal lobe and the occipital lobe was a risk factor for T1D. Among them, interleukin-8 played a mediating role in the mediation of temporal lobe-related functional connectivity on T1D, and programmed cell death 1 ligand 1 played a mediating role in the mediation of temporal lobe on LE. This co-occurrence suggests a close relationship between temporal lobe dysfunction and the development of AIDs [33, 34].

These findings, together with our research, collectively suggest that the CNS has significant potential for immune regulation. Our findings highlight the critical role of functional networks in the frontal lobe, temporal lobe, and cerebellum within the neuro-immune axis (Additional file 1: Table S3). Previous studies have confirmed that both the frontal and temporal lobes can modulate peripheral immunity via the hypothalamic-pituitary-adrenal (HPA) axis [35–37]. Furthermore, the superior cerebellum, including the vermis VI, is an integral component of the central autonomic nervous system network [38]. These support the notion that the regulatory pathways underlying the effects of these brain structures on peripheral immune function may involve both the HPA axis and the autonomic nervous system, encompassing both sympathetic and parasympathetic pathways.

During mediation analysis, we also observed that certain mediators exhibit effects opposite to the predefined IDPs-AIDs causality. For instance, interferon gamma levels exert an opposing effect in the regulation of “aparc-a2009s lh area S-temporal-inf” in RA (Additional file 1: Table S9). In causal mediation analysis, the indirect effect represents the portion of the total exposure-to-outcome association that operates through the specified mediator. A negative indirect effect indicates that the mediator acts in a direction opposite to the overall or direct pathway, often reflecting suppressive, inconsistent, or counteracting mediation. Although immune cells and inflammatory factors showed significant mediating effects in this study, their mediation proportions did not exceed 5%, indicating that these peripheral immune phenotypes only explained a very small portion of the total effect of exposure on AIDS risk. This suggests that peripheral immunity/inflammation is not the main driving factor in the pathogenesis of AIDS, but rather likely acts as a secondary regulatory or concomitant mechanism. This finding is consistent with the multifactorial etiology of AIDS and provides a basis for subsequent intervention strategies focusing on core neuropathology in the brain.

Despite the valuable insights provided by our study, several limitations should be acknowledged to effectively contextualize and interpret the findings. First, our Mendelian randomization analysis was restricted to 91 inflammatory factors and 731 immune cells, thus limiting the generalizability of our findings. Second, due to the limited open access to summary-level GWAS data, the current analysis population was exclusively composed of European participants. Future studies encompassing multiple ethnic groups and larger sample sizes are necessary to confirm our conclusions. Third, we were unable to obtain individual-level associations because the genetic information of the subjects was unavailable. This precluded us from performing subgroup analysis, which may have introduced some biases to our results. Finally, we assumed a linear relationship between exposure and outcome in our MR analysis, potentially overlooking more complex and nonlinear relationships.

## Conclusion

This study employed a comprehensive Mendelian randomization approach to elucidate the causal relationships between brain IDPs and AIDs, providing critical insights into the neuro-immune axis. By pinpointing specific brain regions, such as the frontal lobe and temporal lobe, our findings underscore their crucial roles in linking CNS function with peripheral immune regulation.

## Supplementary Information

Below is the link to the electronic supplementary material.

Supplementary Material 1

## Data Availability

The data that supports the findings of this study are available in the supplementary material of this article.

## Abbreviations

AD: Atopic dermatitis
AIDs: Autoimmune diseases
CI: Confidence interval
CNS: Central nervous system
DMN: Default mode network
DTI: Diffusion tensor imaging
GWAS: Genome-wide association study
HPA: Hypothalamic-pituitary-adrenal
IBD: Inflammatory bowel disease
ICA: Independent Component Analysis
IDPs: Imaging-derived phenotypes
IFNγ: Interferon-γ
IVW: Inverse variance weighted
LE: Lupus erythematosus
MG: Myasthenia gravis
MR: Mendelian randomization
MS: Multiple sclerosis
OR: Odds ratio
PSO: Psoriasis
RA: Rheumatoid arthritis
SLE: Systemic lupus erythematosus
SNP: Single nucleotide polymorphism
T1D: Type 1 diabetes
TNFα: Tumor necrosis factor-α
